# Assessment of Pain Management, Acceptance of Illness, and Adjustment to Life with Cancer in Patients with Nonmuscle Invasive Bladder Cancer

**DOI:** 10.1155/2018/7598632

**Published:** 2018-10-22

**Authors:** Wojciech Krajewski, Małgorzata Mazur, Adrian Poterek, Agata Pastuszak, Urszula Halska, Andrzej Tukiendorf, Joanna Rymaszewska, Romuald Zdrojowy

**Affiliations:** ^1^Department of Urology and Oncological Urology, Wrocław Medical University, Borowska 213 50-556, Wrocław, Poland; ^2^Department of Physiotherapy, Opole Medical School, Katowicka 68, 45-060 Opole, Poland; ^3^Department of Social Medicine, Wrocław Medical University, Pasteura 10, 50-367 Wrocław, Poland; ^4^Department of Psychiatry, Wrocław Medical University, Pasteura 10, 50-367 Wrocław, Poland

## Abstract

**Purpose:**

According to the European Association of Urology bladder cancer is the seventh most commonly diagnosed malignancy in the world's male population. Despite its high incidence, papers evaluating psychological state in those patients' group are lacking. The purpose of the study was to evaluate pain management, disease acceptance, and adjustment to cancer in homogenous group of patients diagnosed with nonmuscle-invasive bladder cancer (NMIBC).

**Methods:**

Group of 252 male patients who were scheduled for NMIBC treatment were prospectively evaluated. Patients fulfilled Acceptance of Illness Scale (AIS), Mini-Mental Adjustment to Cancer (Mini-MAC) and Coping Strategies (CSQ) questionnaires before treatment introduction.

**Results:**

Highest CSQ score was achieved by the coping self-statements subscale (mean=18,37). The catastrophizing subscale score was the lowest (mean=11,24). Place of residence affected results of CSQ statement about pain control. Catastrophizing and coping self-statements strategies were associated with matrimonial status. In the Mini-MAC questionnaire the fighting spirit way of coping had the highest (21,73) and the helplessness-hopelessness subscale had the lowest mean value (13,3). Matrimonial status was strongly associated with anxious preoccupation, fighting spirit, and helplessness – hopelessness way of coping. The mean AIS test score was 28.8. AIS result was influenced by patient's marital status, yet not by education, place of residence, nor any clinical factor.

**Conclusions:**

In the examined group, the level of acceptance of the disease reached values that were slightly higher than the average. It indicated a fairly good adaptation to cancer. Among the methods of coping with cancer, the constructive style is definitely dominant with a high intensity of the fighting spirit strategy. The destructive style of cancer coping reached low values with a low intensity of helplessness/hopelessness strategy. From pain coping strategies, self-statements and praying/hoping were the most commonly chosen ways, whereas catastrophizing was the rarest. Many associations between various questioners' results were also observed.

## 1. Introduction

According to the European Association of Urology (EAU) bladder cancer (BC) is the seventh most commonly diagnosed malignancy in the world's male population [1]. In the European Union the age-standardised incidence rate for men is 19.1 [2]. Nearly three-quarters of all BC patients are diagnosed with nonmuscle invasive BC (NMIBC). Those cases contain tumours that are confined to the bladder mucosa (stage Ta, CIS) or submucosa (stage T1), yet not infiltrating muscular layer of the bladder (stages T2 and higher). Due to nonadvanced stage, correctly managed NMIBCs present relatively good survival rates, however, with high perpetual risk of recurrence or progression occurrence. Thereat, disease-free survival requires lifelong observation with repetitive implementation of cystoscopies and transurethral resections. This can be achieved only with close patient-therapeutic team collaboration and good compliance to diagnostic and treatment programmes. Nevertheless, invasive manipulation on genitourinary tract has negative impact on patients' psychological wellbeing and may aggravate pre-existing psychological problems. This, in many cases, leads to the avoidance or total abandonment of the scheduled disease management and in result to uncontrolled tumour spreading.

Despite the fact that NMIBC represents majority of BC cases, papers evaluating psychological state in those patients' group are lacking. Available studies concerning BC cases apply solely to muscle invasive BC and its extensive mutilating proceedings [3–5].

## 2. Aim

The purpose of the study was to evaluate pain management, disease acceptance, and adjustment to cancer in homogenous group of patients diagnosed with NMIBC. We also analysed the effect of socioeconomic and clinical variables on the above mentioned issues.

## 3. Material and Methods

An institutional ethics committee approved the study and all patients gave written informed consent.

We prospectively evaluated pain management, disease acceptance, and adjustment to cancer in 367 male patients with NMIBC who were scheduled for a transurethral resection of bladder tumour (TURB) procedure. The patients were recruited during 12 months' period (11.2016- 11.2017) in 3 polish centres: the Urology and Urologic Oncology Department of Wroclaw Medical University, Department of General, in the Oncological and Functional Oncology of Medical University of Warsaw, and in the Department of Urology and Oncologic Urology of Lower Silesian Specialistic Wrocław Hospital.

Due to the fact that this analysis is a part of a study analysing TURB influence on patient psychological well-being following exclusion criteria were adopted. Patients with age under 18 years old enable to understand the information about the study or to cooperate with psychological evaluations and taking medications that could affect their mental status were disqualified.

From the initially qualified population (367 pts.) 68 patients, who did not fulfil the questionnaires, were not included in analyses (drop-out). Those, who did not provide or did not correctly fulfil the Mini-Mental Adjustment to Cancer questionnaire (Mini-MAC) (17 pts.) and/or Coping Strategies Questionnaire (CSQ) (49 pts.) but filled completely Acceptance of Illness Scale (AIS) were included in analysis. Finally, data from 252 patients were included in the analysis (AIS correctly fulfilled by 252 pts., Mini-MAC by 235 pts., and CSQ by 201 pts). Additionally, as a part of bigger study, results of other questionnaires were available (252 of Hospital Anxiety and Depression Scale (HADS) and 196 of The Sexual Satisfaction Questionnaire (SSQ) and the simplified International Index of Erectile Function (IIEF-5)).

The questionnaires were completed after admission to the departments, yet, before surgical treatment (transurethral resection of bladder tumour—TURB procedure), in a space that provided privacy. The investigating urologist explained to each patient the purpose of the study, the protection of participant confidentiality, and the participants' freedom to drop out at any time. The study included individuals who gave informed, written consent to participate.

Demographic (education, marital status, and residency), clinical, and psychometric data were collected. Clinical details such as ASA score, body mass index (BMI), nicotine abuse, history of previous TURB, recurrence/year index, previous intravesical chemotherapy, and/or BCG immunotherapy were recorded. Blood and urine laboratory analysis components were also documented as well as tumour features. Pathological stages were evaluated for all the patients according to the TNM staging system.

Psychometric tools used in the study included the Acceptance of Illness Scale, the Mini-Mental Adjustment to Cancer questionnaire, and the Coping Strategies Questionnaire.

### 3.1. Acceptance of Illness Scale

The Acceptance of Illness Scale is a questionnaire designed for measuring any disease acceptance in adult individuals [6]. The tool is constituted of eight statements forming a single scale, each graded on a scale from 1 to 5. The total score of AIS may be between 8 and 40. The low AIS score shows a lack of adjustment to the disease, no acceptance of the condition, and mental discomfort. The high score is indicative of good disease acceptance [7].

### 3.2. Mini-Mental Adjustment to Cancer

The Mini-Mental Adjustment to Cancer (Mini-MAC) is a 29-statements questionnaire, which analyses four methods of cancer coping (anxious preoccupation, fighting spirit, helplessness-hopelessness, and positive re-evaluation). When put together, anxious preoccupation and helplessness-hopelessness form the passive (destructive) style of coping and the fighting spirit and positive re-evaluation form active (constructive) way of coping. Each statement is scored on a four-point scale ranging from 1 (definitely not) to 4 (definitely yes). The points in each strategy are summed separately resulting in final range of 7-28 points. The higher the score, the more intense the behaviour patterns typical for a given coping strategy [8].

### 3.3. Coping Strategies Questionnaire

The Coping Strategies Questionnaire was designed by Rosenstiel and Keefe to assess patient pain coping strategies and their effectiveness in pain alleviation and control [9]. Techniques of coping with pain are combined into six cognitive (diverting attention, reinterpreting pain sensations, catastrophizing, ignoring pain, praying/hoping, and coping self-statements) and one behavioural strategy (increased behavioural activity), which ultimately can be assigned to three components: cognitive coping, diverting attention and undertaking replacement activities, and catastrophizing and hope seeking. Each of 42 statements of CSQ is completed using a 0 (never) to 6 (I always do that) scale. Additionally, two analogously graded statements about pain control and ability to reduce pain are provided. For each strategy, the calculated score is within the range 0 to 36 points. The higher is the score, the greater is the meaning attributed to a given factor in the process of pain coping [7, 9].

In the statistical analysis, Pearson's r linear correlations between continuous outcome variables were calculated. Statistical differences of means between considered groups of patients were estimated based on the t-Student's test and one-way analysis of variance. The computation was performed in the R platform [10].

## 4. Results

The study involved 252 male NMIBC patients (age 18-98, mean 66, and +/- 12), who were scheduled for the TURB procedure because of proven or suspected BC.  Table 1 presents patients' characteristics (Table 1).

The mean scores of each scales are presented in Table 2. When analysing CSQ results it was shown that the highest score in the test was achieved by the coping self-statements subscale (mean =18,37). The catastrophizing subscale scored the lowest (mean = 11,24) (Table 2). When evaluating demographic variables, we found out that place of residence affected results of CSQ statement about pain control. What is more, catastrophizing and coping self-statements strategies were associated with marital status. Clinical and tumour features were also analysed and results are presented in Tables 3 and 4. Tables present only statistically significant associations.

In the Mini-MAC questionnaire the fighting spirit way of coping had the highest (21,73) and the helplessness-hopelessness subscale had the lowest mean value (13,3) (Table 2).

When analysing demographic features, it was shown, that matrimonial status was strongly associated with anxious preoccupation, fighting spirit, and helplessness – hopelessness way of coping. Influence of individual clinical features were minor.

The mean AIS test score was 28.8. Amounts and percent of patients who have chosen answer 4 and 5 are also listed in the Table 2. AIS result was influenced by patient's marital status (Table 3), yet, not by education, place of residence nor any clinical factor.

What is more, Pearson's r linear correlations between questionnaire's scores and risk factors and between each questionnaire's results were performed. Because of the fact that this paper is a part of a bigger study evaluating TURB impact on patients psychological well-being, preoperative scores of SSQ, HADS, and IIEF was also available and therefore analysed. From risk factors only a number of previous TURBs were positively correlated with CSQ ignoring pain strategy. Results of correlations between each questionnaire's scores are presented in Table 5.

In one-way ANOVA analysis CSQ pain reduction subscale result was associated with place of residence with higher score for smaller district. T-Student's test results are presented in Tables 3 and 4.

## 5. Discussion

In the majority of cancer patients, emotions such as anxiety, restlessness, helplessness, and weakness are commonly present. Those negative feelings in addition to long, burdensome, and often mutilating treatment influence significantly patients' quality of life [11, 12].

Being diagnosed with malignant disease is an emotional, psychological, and physical traumatic experience. In the best part of cases it is associated with considerable life changes and necessity to adapt to new circumstances. Many studies prove that these changes are often negative and are connected to stress caused by the diagnosis, fear of the disease, and fear of death.

It is widely known that coping strategies, illness acceptance and adjustment to cancer have a substantial impact on the treatment of malignant disease [7, 13–15]. Hence, it appears vital to identify the cognitive and behavioural aspects that may indorse adaptive functioning of the patient experiencing cancer and pain. These factors may help to understand differences in adjustment between patients, determine their cognitive strengths and weaknesses, identify treatment objectives and predict therapeutic outcomes [16, 17]. In this study we analysed adjustment to cancer, pain coping and disease acceptance in a big homogenous group of male patients diagnosed with NMIBC.

### 5.1. MiniMAC

Coping is an essential process that involves an individual's cognitive and behavioural efforts to manage a stressor [18]. In the case of cancer, coping is the method in which the patient responds to the diagnosis and how one deals with the disease [19]. Coping is a skill that helps patients stabilize their responses to the life-altering diagnosis and its further repercussions [20, 21]. It was proved that the patients' coping strategies have an effect on quality-of-life, psychological well-being, social interactions, and how they integrate the illness into their life. What is more, these coping efforts can persist in the patient's life posttreatment [21]. It was also shown that constructive way of coping with the disease is an important feature allowing for longer survival and better quality of life of the patient [22, 23].

In this study population constructive style of coping with illness scored higher than destructive, with fighting spirit mean score of 21,73 and helplessness-hopelessness of 13,3 points. Those observations are similar to other papers evaluating cancerous patients in polish population [24, 25] especially in patients with gastric, pancreatic, colorectal, or prostate carcinoma [26, 27].

Other authors observed that adopted strategies of coping with disease may change with time from diagnosis and treatment. The change was more evident for constructive models [26, 28, 29]. Szczepańska et al proved that in patients with breast cancer the constructive models are the highest during hospitalization and treatment. In the same time, destructive scores are low. It may be associated with the strong activation of defensive mechanisms in order to eliminate negative emotions. After treatment gradual decrease was observed, however, with stable scores of destructive strategies [27]. In our study we did not assess precise time from primary diagnosis, taking number of previous TURB and recurrence/year index as surrogates. Any statistically significant correlation of those factors and Mini-MAC sores was noted.

It was previously proved, that physical activity has impact on one's attitude to the illness [30, 31]. In the polish population Malicka et al. studied women after breast cancer treatment and concluded that patients who participated in diverse and plentiful physical activities presented higher results in the fighting spirit way of coping. Among many types of physical activity, tourist trips and dance were the most important for quality of life [32]. Unfortunately, those observations cannot be verified in this study population.

When analysing demographic features, it was shown that matrimonial status is strongly associated with almost all types of disease coping. Patients living in active relationships obtained significantly higher score in fighting spirit strategy and lower in both destructive models. It is possible that lone patients have a greater tendency to implement destructive styles of coping. Yet, it was not confirmed in study on females with breast cancer, however some statistically insignificant trends were noticed [25].

In other papers influence of place of residence was noted, with the anxious preoccupation score decreasing along the size of the place of residence [25, 33]. In our population the observation was not confirmed. Also financial income and education influence was observed, having biggest influence on destructive strategies. In both coping models, the mini- MAC scores decreased along with an increase in income and education [33, 34]. In our population the observation for education was not confirmed. Also, analysis of impact of clinical factor did not reveal any significant association.

### 5.2. CSQ

Upwards of half of cancerous patients experience pain associated with the disease. Nevertheless, the pain assessment and its treatment may be very difficult due to its subjectivity, presence of emotional components, and the fact that not only pain intensity but also degree of reaction to stimuli vary from one person to another [35, 36]. Available research showed the complexity of the mechanism of pain creation and the pain dealing strategies selection. It is widely known that the sensation of pain and its level is fundamentally reliant on psychological aspects. Psychological factors play also a vital role in selecting coping strategies by the patient [37–39]. Patients adopt various way in an attempt to fight with pain and the type of chosen method is highly affected by individual convictions [40]. Yet, choosing a coping strategy has a significant influence on the health-related quality of life [41]. It was proved by Jackson et al. that adoption of the helplessness strategy significantly affects the feeling of pain and increase incidence of depression [42]. What was also shown in other papers is that patients who apply passive coping strategies are proved to sense pain as much more intense than those applying active strategies [33, 43, 44].

In this population, coping self-statements and praying/hoping where the most commonly chosen pain coping strategies, whereas catastrophizing was the rarest. Those observations are in accord with other paper on cancerous patients in polish population [9, 33, 37, 45, 46]. Despite the fact that patient's age was proved to influence CSQ outcomes by several other studies, in our study the differences were not statistically significant [47]. Patient's education and financial income impact was also proved in some studies, yet not in this analysis [24, 33]. When analysing place of residence, it was shown that patients from rural areas had significantly higher scores of pain reduction abilities comparing to patients with bigger cities. This is in opposite to other authors [24, 45]. Additionally, association with marital status was observed. The single patients had significantly higher catastrophizing and lower self-statements scores. What is interesting is that increased behavioural activity was shown to be associated with patients nicotinism, with higher scores for patients who smoke cigarettes.

### 5.3. AIS

Malignant disease affects various aspects of one's life and, for that reason, acceptance of the illness is a serious issue for patients with cancer. From a simpler perspective, acceptance of the illness may be considered as an approval to life changes. It is proven that higher level of cancer acceptance prevents negative emotions intensification, is correlated with symptom severity, facilitates acceptance of disease-induced limitations and leads to greater motivation to fight disease [48–50]. From the other side, the lack of consent to restrictions leads to exhaustion and helplessness. Often, as a result of patient's misconceptions, lack of a disease acceptance is a bigger problem than the disease itself [51].

In the analysed population mean score of Acceptance of Illness Scale was 28,8. More than 55% of patients have chosen answers 4 and 5 declaring that one disagrees or definitely disagrees with the AIS statements. This is all indicative for relatively good disease acceptance. Those results seem to be higher when compared with other, noncancerous patients analysed by Juczyński [7]. In his studies analysing diabetic patients, males after myocardial infarction, males with chronic pain, and females diagnosed with migraine scored 24.82, 22.14, 18.46, and 24.23 points, respectively. In patients with malignancies, breast, uterine prostate, and colon cancer patients showed similar levels of acceptance of illness comparing to our population [7, 24, 33, 45, 52, 53].

Negative emotions triggered by malignant disease might make a person feel redundant. What is more, pain, therapeutic proceedings, and/or frequent and prolonged hospitalizations make patient feel submissive, dependent, or incompetent. When analysing meticulously the particular statements scores it could be noted that more than 60% of the study population strongly contradicts the fact feeling unneeded or being dependent on others. Those numbers are similar when comparing to majority of papers on various malignancies in polish population. In contrast, patients with leukaemia scored significantly lower [54].

Evaluation of sociodemographic factors influence on AIS was also performed. In opposite to other authors, we have shown that matrimonial status had significant impact on AIS [25]. Single males had mean 4,3 less AIS points than those living in relationships. Education level and place of residency did not reveal dependency on AIS. It is in concordance with other studies from polish population [52, 55, 56]. In some papers patients' age and financial income are presented to have influence on AIS results [52, 56]. In our population we did not assess the income and did not find any statistical association with age. However, which is different from other papers, the age range in our population is relatively narrow and it can efface the difference.

It has to be kept in mind that the AIS values may differ with time after cancer treatment. [52, 57]. As stated above, number of previous TURB and recurrence/year index were analysed as time-from-diagnosis surrogates. Any statistically significant correlation of those factors and AIS sore was noted. Finally, any other clinical feature was not statistically correlated with the AIS result.

To analyse associations between each questionnaire's results Pearson's r linear correlations were performed. Pre-TURB scores of SSQ, HADS and IIEF were also available and therefore analysed. It was showed that there were strong correlations between various scales, especially between each score within one tool. It suggests consistent structure of questionnaires and appropriate selection of questions. What is worth noticing is that depressive and anxiety HADS scores were strongly negatively correlated with acceptance with illness, pain control, and pain reduction scores and positively with catastrophizing coping strategy. Also, solid correlations between HADS and Mini-MAC scores may be observed, as well as between Mini-MAC and some CSQ scores. This is especially evident when catastrophizing coping strategy is compared to anxious preoccupation and helplessness-hopelessness Mini-MAC strategies.

Data analysis allows assuming that the higher the AIS score, the more often the constructive way of coping is used. AIS was also positively associated with pain control and pain reduction scores, emphasizing earlier observations that patients who accept their illness cope better with the pain and with the disease itself. Opposite conclusions can be drawn for catastrophizing and praying/hoping pain coping strategies as well as for destructive style of disease coping. What is more, SSQ and IIEF scores were strongly linked with AIS scores. This one more time accentuates the fact that good disease acceptance has positive influence on various sections of patient's mental well-being.

### 5.4. Limitations and Strengths

Our study has some limitations. Firstly, there might have been some qualification bias, as we included only patients that were able to fill out surveys completely without the help of third parties. Some were unable to fill the initial questionnaires, which might have resulted from strong anxiety at the admission for operation. For that reasons, the paper possibly does not include a complete cross-section of NMIBC patients. Secondly the study includes only male patients.

Despite limitation our report has some clear strengths. Study was conducted in high-volume oncology-orientated centres, which certifies the quality and repeatability of the procedures. Secondly the study sample was homogenous and significantly bigger than in other papers evaluating other malignancies. Thirdly we present analysis of the problem that was not addressed previously in available literature.

## 6. Conclusion

In the examined group, the level of acceptance of the disease reached values that were slightly higher than the average. It indicated a fairly good adaptation to cancer. Among the methods of coping with cancer, the constructive style is definitely dominant with a high intensity of the fighting spirit strategy. The destructive style of cancer coping had reached low values among the respondents with a low intensity of helplessness/hopelessness strategy. From pain coping strategies, self-statements and praying/hoping were the most commonly chosen ways, whereas catastrophizing was the rarest. Many associations between various questioners' results were also observed.

In modern medicine, the proceedings of medical personnel should include both proper therapy and ensuring the best possible patients' psychological well-being. The results of this study emphasise the significance of personalized medicine based on the work of a multidisciplinary team. Therapeutic groups ought to include individuals who routinely evaluate patients' mental state and patients' adaptation to one's health situation.

## Figures and Tables

**Table 1:** Patients' baseline characteristics.

**Clinical characteristic**	**Strata**	**Prevalence n; (**%**)**
Age	<65	95 (37,7%)
	65-70	68 (27%)
	70-75	36 (14,3%)
	>75	53 (21%)

ASA	1	60 (23,8%)
	2	141 (55,9%)
	3	51 (20,3%)

BMI	≤25	72 (28,6%)
	25,1-30	112 (44,4%)
	30,1-35	40 (15,9%)
	>35	17 (6,7%)
	missing	11 (4,4%)

Nicotine abuse	yes	115(45,6%)
	no	126 (50%)
	missing	11 (4,4%)

Prior TURB	0	101 (40%)
	1	72 (28,6%)
	2	37 (14,7%)
	≥3	42 (16,7%)

Rec/year	0	101 (40%)
	<1	81 (32,2%)
	>1	70 (27,8%)

Preoperative chemotherapy	yes	7 (2,8%)
	no	242 (96%)
	missing	3 (1,2%)

Preoperative BCG immunotherapy	yes	34 (13,5%)
	no	216 (85,7%)
	missing	2 (0,8%)

Preoperative hematuria	yes	91 (36,1%)
	no	150 (59,5%)
	missing	11 (4,4%)

Preoperative pyuria	yes	101 (40,1%)
	no	140 (55,5%)
	missing	11 (4,4%)

Education	primary	33
	secondary/vocational	45
	higher	17
	missing	5

Marital status	single	11
	coupled	59
	missing	5

Place of residence	village	62
	City <50k	33
	City >50k	5

Number of tumors	1	162 (64,3%)
	2	39 (15,5%)
	≥3	51 (20,2%)

Tumor size	<1cm	91 (36,1%)
	1-3cm	108 (42,9%)
	>3cm	53 (21%)

Tumour stage (T)	0	35 (14%)
	A	111 (44%)
	1	56 (22%)
	2	32 (12,1%)
	Other than UCC	6 (2,4%)
	missing	4 (1,6%)
	CIS	8 (3,2%)

High grade tumour	yes	87 (34,5%)
	no	161 (63,9%)
	missing	4 (1,6%)

CIS concomitants	yes	35 (13,9%)
	no	213 (84,5%)
	missing	4 (1,6%)

Rec: recurrence; ASA: Physical Status Classification System; BMI: Body Mass Index; TURB: transurethral resection of the bladder; BCG: Bacillus Calmette-Guérin; CIS: Carcinoma in situ. Missing data only when indicated.

**Table 2:** CSQ, Mini-MAC and AIS scores. In AIS subscale the number of patients who have chosen answers 4 and 5 is listed.

**CSQ**	**Mean**	**Standard deviation**
Diverting attention	16,13	8,16
Catastrophizing	11,24	9,2
Reinterpreting pain sensations	12,3	8,88
Ignoring pain	15,5	9,17
Praying/hoping	17,7	9,98
Coping self statements	18,37	8,78
Increased behavioural activity	17,24	8,87

**Mini-Mac**		

Anxious preoccupation	15,71	4,96
Fighting spirit	21,73	4,01
Helplessness – hopelessness	13,3	4,59
Positive re-evaluation	20,65	3,64

**AIS**	**28,8**	**7,54**

I find it difficult to adjust to disease-induced limitations	3,53	132 (52,4%)
Because of my condition I cannot do what I like most	3,51	133 (52,8%)
My disease makes me feel redundant at times	3,74	156 (61,9%)
Health issues make me more dependent on others than I wish I were	3,5	131 (52%)
My disease makes me a burden for my family and friends	3,73	152 (60,3%)
My condition makes me feel incompetent	3,71	156 (61,9%)
I will never be as self-sufficient as I would like to	3,61	142 (56,3%)
I believe that people who spend a lot of time with me are embarrassed because of my disease	3,62	143 (56,7%)

**Table 3:** T-Student's Test analysis between matrimonial status and questionaries' scores.

**Scale**	**Matrimonial Status**	**Single**	**Married**	**P-Value**
**CSQ catastrophizing**	Mean	13.9	10.2	0.01
	SD	10.9	8.3	

**CSQ self statements**	Mean	15.6	19.5	0.005
	SD	8.2	8.8	

**AIS**	Mean	25.7	30.0	0.001
	SD	7.9	7.1	

**mMac anxious preoccupation**	Mean	17.4	15.1	0.002
	SD	5.1	4.8	

**mMac fighting spirit**	Mean	20.4	22.2	0.003
	SD	4.4	3.8	

**mMac helplessness**	Mean	14.9	12.8	0.002
	SD	5.2	4.3	

mMac: Mini-Mental Adjustment to Cancer. Only statistically significant and borderline correlations are listed.

**Table 4:** T-Student's Test analysis between risk factors and questionaries' scores.

**Scale**	**Risk factor**			**P-Value**
	**Nicotine**	No	Yes	

**CSQ behavioural activity**	Mean	18.51	15.66	0.0232
	SD	9.03	8.53	

	**Intravesical chemotherapy**	No	Yes	

**CSQ reinterpreting**	Mean	12.53	5.71	0.0463
	SD	8.82	9.43	

	**Pyuria**	No	Yes	

**CSQ pain control**	Mean	3.72	3.27	0.0383
	SD	1.42	1.55	

Only statistically significant and borderline correlations are listed.

**Table 5:** Statistically significant Pearson's r linear correlations (p<0.05) between psychological scales.

	**CSQ pain reduction**	**CSQ reinterpreting**	**CSQ catastrophizing**	**CSQ ignoring pain**	**CSQ praying**	**CSQ self statements**	**CSQ behavioural activity**	**AIS**	**mMac anxious preoccupation**	**mMac fighting spirit**	**mMac helplessness**	**mMac re-evaluation**	**SS**	**HADS anxiety**	**HADS depressive**	**IIEF**
**CSQ pain control**	0.81	0.21	-0.46	0.26	-0.21	0.37	0.31	0.46	-0.46	0.30	-0.47	0.34	-	-0.4	-0.55	-
**CSQ pain reduction**	-	-	-0.44	-	-0.22	0.23	0.27	0.50	-0.44	0.30	-0.45	0.32	0.23	-0.43	-0.55	-
**CSQ diverting attention**		0.54	0.24	0.52	0.59	0.48	0.68	-	-	-	-	0.14	-	-	-	-
**CSQ Reinterpreting**			0.25	0.68	0.34	0.51	0.53	-	-	0.27	-	0.21	-	-	-	-
**CSQ catastrophizing**				-	0.38	-	-	-0.43	0.56	-0.23	0.53	-0.22	-0.34	0.5	0.56	-
**CSQ ignoring pain**					0.33	0.68	0.43	-	-	0.23	-	0.18	0.25	-	-	-
**CSQ praying**						0.40	0.35	-0.18	0.27	-	0.24	-	-	-	0.24	-
**CSQ self statements**							0.48	0.18	-0.17	0.24	-0.25	0.26	0.29	-	-0.31	-
**CSQ behavioural**								-	-0.16	0.22	-0.20	0.25	-	-	-0.3	-
**AIS**									-0.42	0.38	-0.49	0.30	0.47	-0.4	-0.57	0.42
**mMac anxious preoccupation**										-0.30	0.72	-0.28	-0.34	0.66	0.52	-
**mMac fighting spirit**											-0.37	0.68	0.32	-0.41	-0.41	-
**mMac helplessness**												-0.38	-0.36	0.5	0.62	-0.2
**mMac re-evaluation**													-	-0.33	-0.37	-

mMac: Mini-Mental Adjustment to Cancer; SS: Sexual Satisfaction Questionnaire score; IIEF: International Index of Erectile Function score. Only statistically significant and borderline correlations are listed.

## Data Availability

The data used to support the findings of this study are available from the corresponding author upon request.
